# Odorant Receptor 51E2 Agonist β-ionone Regulates RPE Cell Migration and Proliferation

**DOI:** 10.3389/fphys.2017.00888

**Published:** 2017-11-30

**Authors:** Nikolina Jovancevic, Soumaya Khalfaoui, Markus Weinrich, Daniel Weidinger, Annika Simon, Benjamin Kalbe, Marcus Kernt, Anselm Kampik, Günter Gisselmann, Lian Gelis, Hanns Hatt

**Affiliations:** ^1^Cell Physiology, Ruhr-University Bochum, Bochum, Germany; ^2^Ophthalmology, Ludwig Maximilian University of Munich, Munich, Germany

**Keywords:** GPCR, gene expression, cell signaling, calcium imaging, cell proliferation

## Abstract

The odorant receptor 51E2 (OR51E2), which is well-characterized in prostate cancer cells and epidermal pigment cells, was identified for the first time as the most highly expressed OR in human fetal and adult retinal pigment epithelial (RPE) cells. Immunofluorescence staining and Western blot analysis revealed OR51E2 localization throughout the cytosol and in the plasma membrane. Additionally, immunohistochemical staining of diverse layers of the eye showed that the expression of OR51E2 is restricted to the pigment cells of the RPE and choroid. The results of Ca^2+^-imaging experiments demonstrate that activation of OR51E2 triggers a Ca^2+^ dependent signal pathway in RPE cells. Downstream signaling of OR51E2 involves the activation of adenylyl cyclase, ERK1/2 and AKT. The activity of these protein kinases likely accounts for the demonstrated increase in the migration and proliferation of RPE cells upon stimulation with the OR51E2 ligand β-ionone. These findings suggest that OR51E2 is involved in the regulation of RPE cell growth. Thus, OR51E2 represents a potential target for the treatment of proliferative disorders.

## Introduction

The retinal pigment epithelium (RPE), a monolayer of pigmented polarized cells, is located between the choroids and the neural retina and represents a part of the blood-retina barrier (Rizzolo, 1997; Marmorstein, 2001). The RPE performs a variety of important functions that are essential for visual perception, such as light absorption, transepithelial transport, isomerization of all-trans to 11-cis retinal, secretion, and phagocytosis (Steinberg, 1985; Miller and Edelman, 1990; Bok, 1993; Stalmans and Himpens, 1997; Baehr et al., 2003; Besch et al., 2003; Strauss, 2005). Most of these functions are controlled by the intracellular Ca^2+^ level, which, in turn, is regulated via G protein-coupled receptors (GPCR) (Wimmers et al., 2007). The genes coding for the odorant receptors (OR) were first demonstrated to be expressed in the olfactory epithelium of the rat, where they provide the elevation of intracellular calcium upon odor stimulation. These genes also represent the largest GPCR family in the human genome (Buck and Axel, 1991). OR expression is found in various human tissues outside the nose, such as the prostate, lung, liver, skin and testis (Feldmesser et al., 2006; Zhang et al., 2007; Flegel et al., 2013). The physiological roles of these ectopically expressed ORs are the subject of ongoing research. The few studies investigating their function show that the activation of human ORs leads to, for example, secretion processes or influences the cell proliferation such as OR51E2 in prostate cancer cells or in melanocytes (Spehr et al., 2003; Braun et al., 2007; Neuhaus et al., 2009; Veitinger et al., 2011; Sanz et al., 2014; Kang et al., 2015; Kim et al., 2015; Gelis et al., 2016; Kalbe et al., 2016b; Manteniotis et al., 2016b; Tsai et al., 2017; Weber et al., 2017). The various physiological functions depend on the tissue and respective OR. In the olfactory sensory neurons, ORs mediate a calcium influx, resulting in the generation of action potentials, which provide a crucial step that leads to olfactory perception (Nakamura and Gold, 1987). The activation of ectopically expressed ORs results, in most cases also, in an increase of the intracellular Ca^2+^ level, but this is not always directly linked to the observed function. Interestingly, a recent transcriptome analysis revealed the cell specific expression of ORs in the human neural retina (Jovancevic et al., 2017b). In addition to the human neural retina, ORs were identified in the human fetal RPE. Moreover, Ma and colleagues identified a mutation in an OR gene expressed in stem cell-derived human RPE cells that is associated with the autosomal dominant retinitis pigmentosa (Ma et al., 2015). However, subsequent commentaries on this study point out the limits of the data obtained by whole-exome sequencing (Zhang and Huang, 2015; Sharon et al., 2016). Early in their development, RPE cells undergo a terminal differentiation, resulting in a minimal proliferation capacity throughout normal life. However, RPE cell proliferation can be induced in disease conditions by a variety of growth factors (Stern and Temple, 2015). The activation of proliferation leads to a repair of RPE layer defects. However, an enhanced proliferation and migration could also be pathological and lead to proliferative vitreoretinopathy, a common cause of visual loss (Qiu et al., 2013).

The purpose of the present study was to investigate the ectopic expression of ORs in the human RPE and the effects of OR activation on intracellular Ca^2+^ level and physiological processes, such as migration and proliferation.

## Materials and methods

### Cell culture

Primary retinal pigment epithelial cells from different human donors (3–10 h postmortem) without any history of eye disease were obtained from the Eye Bank of Ludwig Maximilian University and were prepared as previously described (Kernt et al., 2009). We followed the guidelines of the declaration of Helsinki, patients provided informed consent to the scientific use of the explanted tissue, and the study was approved by the local ethics boards of the clinical and the experimental study contributors (Nr. 331-09). RPE cells were maintained in DMEM (Gibco®, Life Technologies) supplemented with 10% FBS and 100 units/ml penicillin and streptomycin at 37°C in a 5% CO_2_ humidified atmosphere.

### Transcriptome analysis

For the transcriptome analysis, the RNA from human RPE cells was isolated using the RNeasy Plus Mini Kit (Qiagen, Hilden, Germany) according to the manufacturer's protocol, including the DNaseI digestion. The mRNA isolation from the total RNA and sequencing analysis were performed by *GENterprise* Genomics (Mainz, Germany) using the Illumina sequencing platform as paired end (RPE1-2) or single reads (RPE3). The datasets are available under the following NCBI Sequence Read Archive accession numbers: SRR6253241, SRR6253242, SRR6253243. We analyzed the mRNAseq data as previously described (Flegel et al., 2013). The raw sequence data were aligned to the human reference genome hg19 using TopHat (Trapnell et al., 2009). Bowtie, the ultra-fast short-read mapping program, served to arrange the alignment (Langmead et al., 2009). The BAM-files were sorted and indexed using the Samtools software package (Li et al., 2009). The FPKM (fragments per kilobase of exon per million fragments mapped) values were calculated using Cufflinks (Trapnell et al., 2010). We reanalyzed previously published raw data in the same manner to compare with the data newly generated for this study. We used datasets from retina supporting tissue (RPE/Choroid/Sclera) (Li et al., 2014) and from the human fetal retinal pigment epithelium samples that were available in the NCBI SRA archive under the following accession numbers: retina supporting tissue (SRR1067930, SRR1067934, SRR1067937, SRR1067940) and human fetal retinal pigment epithelium (SRR447138, SRR786439). The datasets were summarized, and the expression data are presented as the means of the FPKM values (mFPKM). The neural retina raw data were taken from an earlier study (Jovancevic et al., 2017b). All the datasets were equivalently analyzed with the same parameters. The datasets were visualized and investigated by the Integrative Genomic Viewer (http://software.broadinstitute.org/software/igv/) for proving sequence alignments and for the correct mapping of reads for the top expressed genes. We determined a cutoff value of 0.3 FPKM for OR expression as described in Jovancevic et al. (2017b).

While the raw data analysis was performed on a Linux based computer, further calculations were carried out with Microsoft Excel® (Microsoft, WA, USA) and SigmaPlot 12.3 (Systat Software Inc., San Jose, CA, USA).

### Reverse transcription polymerase chain reaction

The total RNA from human RPE cells was reversely transcribed using the iScript cDNA Synthesis Kit (Bio-Rad Laboratories, Hercules, CA, USA) according to the manufacturer's instructions. The equivalent of ~50 ng of RNA was used for each of the RT-PCR experiments. The PCR was performed under standard PCR-conditions with the Mastercycler ep Gradient S (Eppendorf, Hamburg, Germany) (20 μl total volume, 40 cycles: 95°C, 59°C, 72°C, 45 s each). All experiments were conducted in triplicate. The primers used for RT-PCR were as follows: OR51E2 (5′-actgccttccaagtcagagc-3′ and 5′-cttgcctcccacagcctg−3′), PMEL (5′-gtggtcagcacccagcttat-3′ and 5′-gaggagggggctgttctcac-3′), RLBP1 (5′-gctgctggagaatgaggaaactc-3′ and 5′-ggctggtggatgaagtggat-3′), GNAL (5′-cagaccaggac-ctcctcaga-3′ and 5′-agggactctctcagcctgtt-3′), ADCY3 (5′-aaggattcaaccctgggctc-3′ and 5′-tccagcgtcgcatctcatag-3′), CNGA2 (5′-atctccttgccgatgtccc-3′ and 5′-tacatgcagttccgaaaggtca-3′), CNGA4 (5′-gaggtgctgagcgagtatcc-3′ and 5′-cagccgttcaatgcggtaag-3′) and CNGB1 (5′- cgtagagaaggtgatcccgc-3′ and 5′- gtctgaggcagcacctgtag-3′).

### Antibodies

The following primary antibodies were used: custom-made rabbit polyclonal antibody against OR51E2 (Eurogentec; epitope: ISCDKDLQAVGGK); mouse monoclonal antibody against glycerinaldehyde-3-phosphate-dehydrogenase (GAPDH; cat. no. #ab9485; Abcam); rabbit monoclonal antibody against PCNA (cat. no. #ab18197; Abcam); polyclonal rabbit anti-Gα_s/olf_ antibody (cat. no. #sc-383; Santa Cruz Biotechnology, Dallas, Texas; USA), polyclonal rabbit anti-adenylyl cyclase III antibody (cat. no. #sc-588; Santa Cruz Biotechnology); rabbit monoclonal antibody against phospho-AKT (cat. no. #4060), AKT (cat. no. #4691), phospho-ERK1/2 (cat. no. #4370) and ERK1/2 (cat. no. #4695) (Cell Signaling Technology, Danvers, Massachusetts, USA); secondary goat-anti-rabbit and goat-anti-mouse antibodies conjugated to Alexa Fluor 546 or Alexa Fluor 488 (Life Technologies).

### Immunocytochemistry

RPE cells were seeded on coverslips and maintained as described above and human retina normal tissue slides were purchased from Abcam. The specimens were fixed by incubation in 4% paraformaldehyde at 4°C for 30 min. Afterwards, the cells were washed and permeabilized in PBS+Triton X-100 (PBST). Blocking was performed in PBST+1% gelatin and 5% goat serum for 1 h at room temperature. The cells were then incubated overnight with the primary antibody in PBST+1% gelatin and 2% goat serum at 4°C. For visualization, secondary fluorescent anti-rabbit/mouse IgG antibodies (Life Technologies; 1:1,000 dilution) and 40,6-diamidino-2-phenylindole (DAPI) were used. MaxBlock Autofluorescence Reducing Reagent Kit (Dianova, Hamburg, Germany) was used to reduce potential autofluoresence signal according manufacturer's instruction. Micrographs were taken by using a LSM510 Meta confocal microscope (Zeiss, Jena, Germany).

### Cell surface protein isolation

Biotinylation and isolation of cell surface proteins for Western blotting analysis were performed using the PierceTM Cell Surface Protein Isolation kit according to the manufacturer's instructions (Thermo Fisher Scientific, Waltham, Massachusetts, USA).

### Western blot

RPE cells were homogenized in lysis buffer (50 mm Tris HCl, pH 7.4, 150 mm NaCl, 1 mm EDTA, 1% Triton X-100) with Complete® protease inhibitor mixture and PhosSTOP^TM^ (Roche, Basel, Switzerland) using the Precellys®24 (Bertin Technologies, Montigny-le-Bretonneux, France) and Precellys Ceramic Kit 1.4/1.8 (peglab, Erlangen, Germany). Samples were loaded onto a SDS gel and Western blot analysis was performed as described by Gelis et al. (2016).

### Detection of protein phosphorylation

Cells were grown in T25 flask until reaching 70–80% confluence and afterward treated with the appropriate concentration of β-ionone or solvent only (0.1% DMSO) for 10 or 30 min in a humidified incubator at 37°C. After a washing step with PBS^−/−^, protein isolation was performed as described under “Western Blot.” Detection of relative phosphorylation levels of specific kinases was conducted with the Proteome Profiler Human Phospho-Kinase Array Kit (R&D Systems, Minneapolis, Minnesota, USA) according to manufacturer's protocol or by Western blot with phospho-specific antibodies according to manufacturer's instructions. Detection was done as described in Gelis et al. (2016). The protein levels were quantified using the Java-based ImageJ 1.46 software (Schneider et al., 2012) and the relative pixel intensities of odorant-stimulated samples were normalized to the relative pixel intensities of DMSO-treated samples.

### Ca^2+^ imaging

RPE cells were incubated for 30 min at 37°C in loading buffer (pH 7.4) containing Ringer's solution (125 mM NaCl, 5 mM KCl, 1 mM MgSO_4_, 2.5 mM CaCl_2_, 1 mM KH_2_PO_4_, 10 mM NaHCO_3_ and 20 mM HEPES) and 7.5 μM Fura-2-AM (Life Technologies). After removal of extracellular Fura-2 by washing with Ringer's solution, ratiofluorometric Ca^2+^ imaging was performed using a Zeiss inverted microscope equipped for ratiometric imaging and a Polychrome V monochromator (TILL Photonics, Graefelfing, Germany). Cells were visualized with a 20× objective (UPLSAPO, Olympus, Tokio, Japan). Images were acquired in randomly selected fields of view at 0.5 Hz and integrated fluorescence ratios (f340/f380) were measured using TILLvisION software (TILL Photonics). Odorant was pre-diluted in DMSO and then diluted in Ringer's solution to the final concentration, so that the DMSO concentration did not exceed 0.1% (v/v), which is tolerated by RPE cells. SQ 22,536 and other substances (Sigma-Aldrich, St. Louis, Missouri, USA) were pre-diluted in DMSO unless otherwise by the manufacturer indicated. Basic statistical analysis was performed in Microsoft Excel and SigmaPlot.

### cAMP assay

Fifty to seventy percent confluent RPE cells were stimulated for 30 min with different concentrations of β-ionone, forskolin (10 μM) or solvent only. To detect the cAMP levels of stimulated cells, the cAMP-Glo^TM^ Assay (Promega, Madison, Wisconsin, USA) was used according to the manufacturer's instructions.

### Cell proliferation assay

RPE cells were seeded in 96-well plates at density of 5 × 10^3^. After 24 h at 37°C with 5% CO_2_ cells were stimulated with different concentration of β-ionone or solvent DMSO (control) alone in DMEM. Cell proliferation was investigated after 5 days using the CyQUANT cell proliferation assay kit (Life Technologies). For the visualization of proliferating cells via PCNA staining, cells were treated for 5 days with β-ionone (10 and 100 μM) or solvent only. Afterward, cells were stained with anti-proliferating cell nuclear antigen (PCNA) antibody (1:500) as described under “Immunocytochemistry” and Alexa Fluor 546 phalloidin (Life Technologies; 1:200).

### Gap closure assay

Confluent RPE cells grown in monolayers were scratched using a sterile pipette tip and treated with β-ionone or solvent DMSO (control) in DMEM and DMEM+5% FBS (positive control) for 48 h at 37 °C with 5% CO_2_. The residual overgrowing gap of the migrating cells at 12, 24, and 48 h was measured and quantified relative to the initial scratch area (0 h) with the TScratch software (www.cse-lab.ethz.ch).

### Statistical analysis

Statistical analyses were performed with Microsoft Excel and Sigma Plot 12. All results were tested for normality (Shapiro–Wilk test) and equal variance. The significance levels were calculated with a two-tailed unpaired *t*-test, and significant values classified as ^*^p ≤ 0.05, ^**^p ≤ 0.01 and ^***^p ≤ 0.005. The dose–response curve and the EC_50_ value were calculated using the 3-parameter *Hill* equation. The data were represented as the mean ± SEM (standard error of the mean) from at least three independent experiments.

## Results

### Olfactory receptor 51E2 is expressed in the human retinal pigment epithelium

**Figure 1:**
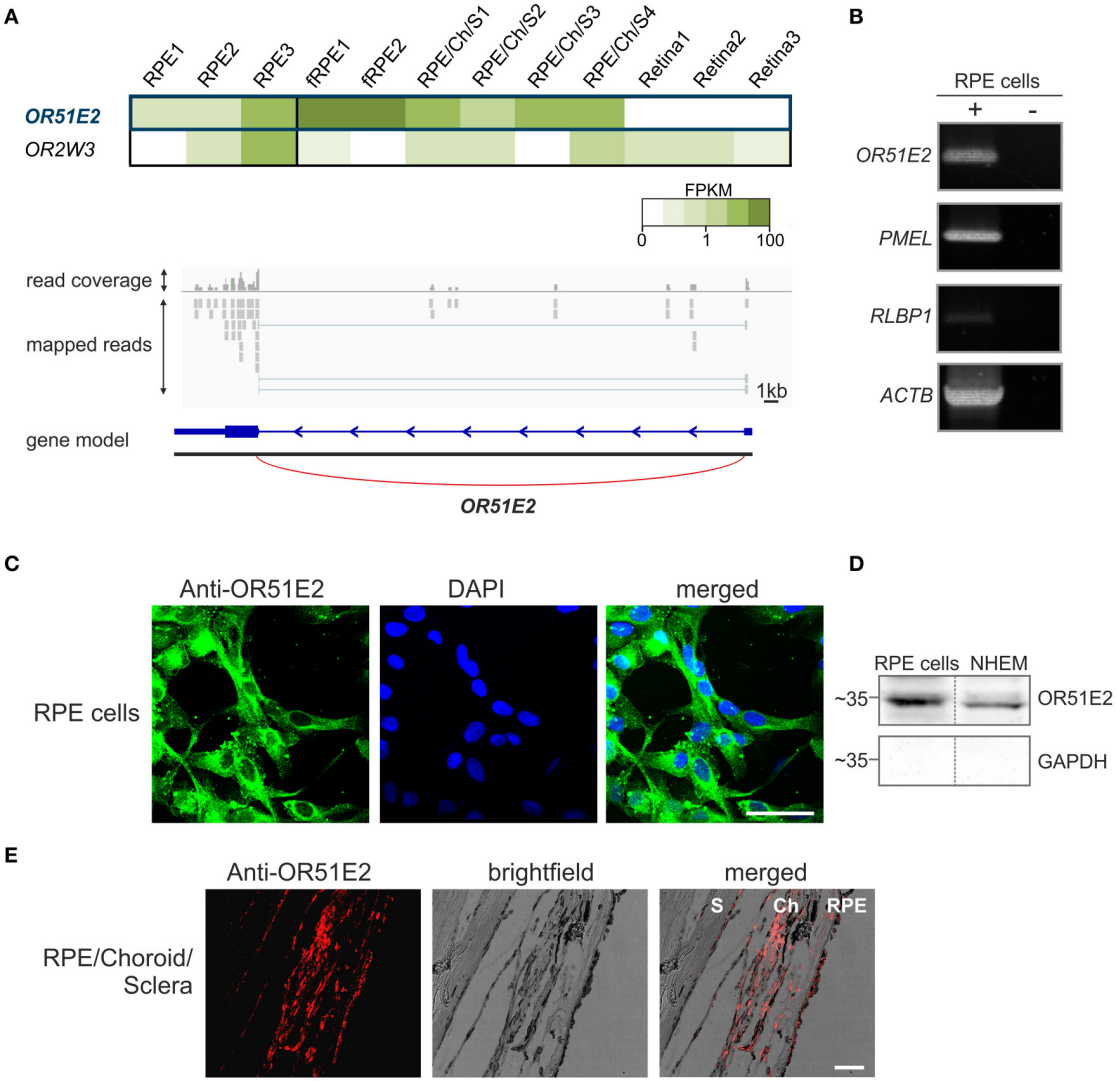
OR51E2 is expressed in human retinal pigment epithelial cells. **(A)** The heat map shows the FPKM values for the most abundant ORs (mean FPKM > 0.3) in three whole RPE samples compared to fetal RPE (fRPE), retina supporting tissue (RPE/Choroid/Sclera) and the three neural retina samples (upper panel). The darker colors indicate higher FPKM values and white indicates the absence of any detectable transcripts. Representation of the read coverage of the *OR51E2* transcripts detected in primary RPE cells and visualized by the Integrative Genomic Viewer (lower panel). The gray segments indicate the reads that were mapped onto the reference genome. The gene is indicated by the blue bars (exon), and thin line (intron) with an arrowhead that shows the reading direction. Read coverage is shown above (detected and mapped counts/bases at each respective position). **(B)** Detection of *OR51E2* transcripts in primary RPE cells by RT-PCR. Gel electrophoresis of amplicons from primary RPE cell cDNA (+) and no reverse transcriptase cDNA controls (–) to exclude genomic DNA contamination. Premelanosome protein (*PMEL*) and Retinaldehyde-binding protein 1 (*RLBP1*) expression identifies RPE cells. The amplification of β-actin (*ACTB)* served as a control for cDNA quality. **(C)** Immunofluorescence confocal micrographs of RPE cells labeled with an OR51E2 specific antibody (green) and DAPI to visualize the nuclei (blue). An intracellular staining of OR51E2 can be observed. The bar indicates 50 μm. **(D)** Plasma membrane localization of OR51E2 in RPE cells verified by surface biotinylation and detection by Western blotting. A representative Western blot of biotinylated membrane of the RPE cells and melanocytes (NHEM), as a positive control, is shown. The cytosolic protein GAPDH served as a control for the enrichment of cell surface proteins and lack of cytosolic proteins. **(E)** Immunofluorescence confocal micrographs of a histological section of RPE, choroid (Ch) and sclera (S) co-labeled with an OR51E2-specific antibody (red). The bar indicates 50 μm.

To generate a more complete view of OR expression in the human RPE, we used mRNA sequencing to characterize the OR expression profile of primary RPE cells from three different healthy donors (Supplementary Table S1) and compared them to reference tissue samples (self-generated data sets of neural retina). In addition, we reanalyzed publicly available data sets from fetal RPE and neural retina supporting tissues. According to our analyses, *OR51E2* was the highest expressed OR transcript in human adult RPE cells and fetal RPE as well as in neural retina supporting tissue, consisting of RPE, choroid and sclera. In the human neural retina, *OR51E2* transcripts were not detectable (Figure 1A). Via the Integrative Genomic Viewer, we confirmed the gene expression of *OR51E2* by the detection of the expression of a non-translated upstream exon (Figure 1A). The detection of annotated 5'UTRs and the corresponding exon-spanning reads is a good proof of the presence of the OR transcript (Flegel et al., 2015a,b). In addition to the *OR51E2* transcript, only one further olfactory receptor, more precisely *OR2W3*, was clearly detectable in two out of the three RPE samples. Apart from the expression level, OR51E2 represent an interesting target for the characterization of an OR in RPE cells because it is also one of the few human ORs for which ligands (β-ionone, short chain fatty acids and androstenone derivatives) have been identified (Neuhaus et al., 2009; Saito et al., 2009). Therefore, we focused on investigating the function of OR51E2 in primary RPE cells. First, we validated the results of the mRNAseq analyses using RT-PCR and detected the transcript expression of *OR51E2* in primary human RPE cells (Figure 1B, Supplementary Figure S1). To confirm the protein expression of OR51E2, immunofluorescence staining of the primary RPE cells with specific custom-made antibodies was performed. The antibody specificity was demonstrated by co-immunocytochemical staining of Hana3A cells heterologously expressing rho-tagged OR51E2 (Gelis et al., 2016; Massberg et al., 2016). OR51E2 was predominantly located in the cytosol (Figure 1C). To deduce if OR51E2 was expressed at the plasma membrane in a low amount, which may be difficult to detect, we analyzed cell surface preparations of primary RPE cells by Western blot analysis. Here, we clearly detected protein localization at the plasma membrane of primary RPE cells (Figure 1D, Supplementary Figure S6). Primary melanocytes served as the positive control for the detection of OR51E2 protein abundance (Gelis et al., 2016). Immunohistochemical analyses of histological sections of human RPE, choroid and sclera further confirmed OR51E2 protein expression in the RPE (Figure 1E). Moreover, OR51E2 was also identified in the choroid, another pigment layer of the eye.

### OR51E2 activation in primary RPE cells

**Figure 2:**
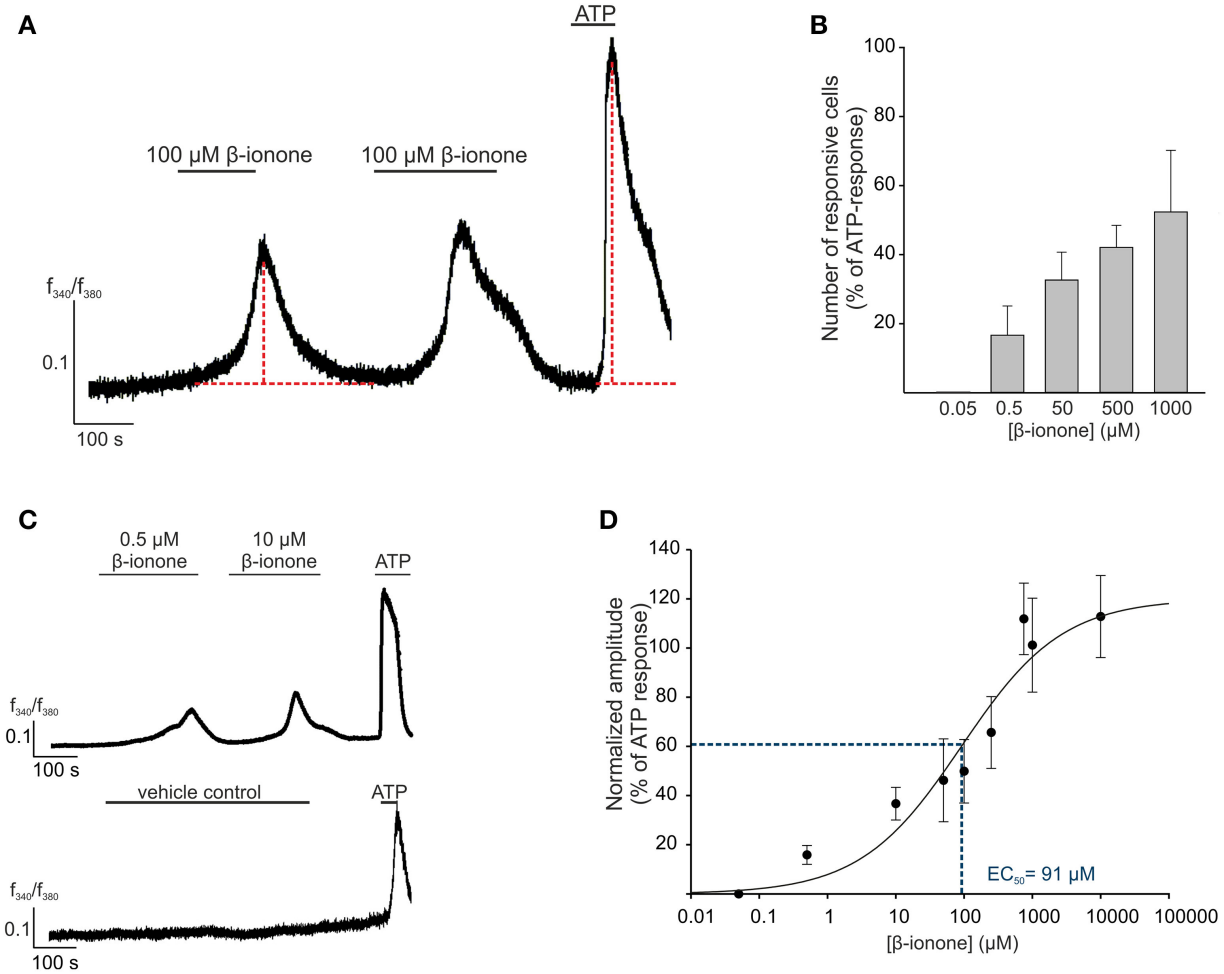
OR51E2-agonist induces Ca^2+^ signals in human RPE cells**. (A)** Representative Ca^2+^ imaging trace of a primary RPE cell. Horizontal bars indicate time and duration of stimulus application. β-ionone (100 μM) application induces a transient rise in intracellular Ca^2+^ in the individual cells. ATP (100 μM) served as the positive stimulus to control cell viability. Cytosolic Ca^2+^ levels were monitored as an integrated f_340_/f_380_ fluorescence ratio, which is expressed as a function of time. In prolonged stimulation, the maximal signal amplitude was reached after a 3 min application of β-ionone. β-ionone induced transient Ca^2+^ signals in primary RPE cells upon repetitive stimulation. **(B)** The β-ionone-induced Ca^2+^ increase is dose-dependent. β-ionone was applied at different concentrations to ensure the maximal number of responsive cells. The number of responsive cells was normalized to the number of ATP-responsive cells [positive control (*n* = 5–7)]. **(C)** Representative Ca^2+^ imaging trace of a Fura-2-loaded human RPE cells. Application of β-ionone induces intracellular Ca^2+^ increase in a dose-dependent manner (upper panel). Application of the solvent (0.1% DMSO) did not result in any changes in cytosolic Ca^2+^ level (lower panel). **(D)** Dose-response curve of the β-ionone-induced Ca^2+^ signals. The signal amplitude, at the first application, was normalized to the amplitude evoked by ATP and is displayed as a function of the applied β-ionone concentration. The EC_50_ value was 91 μM. (*n* = 5–7).

The activation of OR51E2 by its agonist β-ionone induces a rise in cytosolic Ca^2+^ level in epidermal melanocytes, melanoma cells and prostate cancer cells (Neuhaus et al., 2009; Gelis et al., 2016, 2017). As a first step in the functional characterization of OR51E2 in RPE cells, we analogously investigated the effects of short-term (2–5 min) β-ionone stimulation on the intracellular Ca^2+^ levels in primary RPE cells via the Ca^2+^ imaging technology. Stimulation of Fura-2-loaded RPE cells with β-ionone resulted in an increase in intracellular Ca^2+^ concentration with no sensitization of the signal observed in the repetitive stimulation. However, during the whole application of β-ionone, the intracellular Ca^2+^ concentration reaches its maximum and decreases to the cell's basic Ca^2+^ level again (Figure 2A). The β-ionone-induced cytosolic Ca^2+^ response was dose-dependent in amplitude and number of activated cells (Figures 2B–D). The EC_50_ value was 91 μM, and the threshold concentration to trigger a cellular response by β-ionone was under 10 μM (Figures 2C,D). Further OR51E2 agonists also evoked a Ca^2+^ response in RPE cells, whereas compounds that were inactive on the heterologously expressed OR51E2, such as valeric acid, did not affect the intracellular Ca^2+^ level (Supplementary Figure S2). The solvent (0.1% DMSO) did not exhibit any effect when applied alone (Figure 2C). To determine whether the β-ionone-induced Ca^2+^ signals were mediated by OR51E2, we tried to perform RNA silencing experiments with OR51E2-targeted siRNA. However, because RPE cells failed to undergo transfection, we were not able to clearly demonstrate that the β-ionone-mediated effects depend on the activation of OR51E2.

### β-ionone-induced signaling in RPE cells

**Figure 3:**
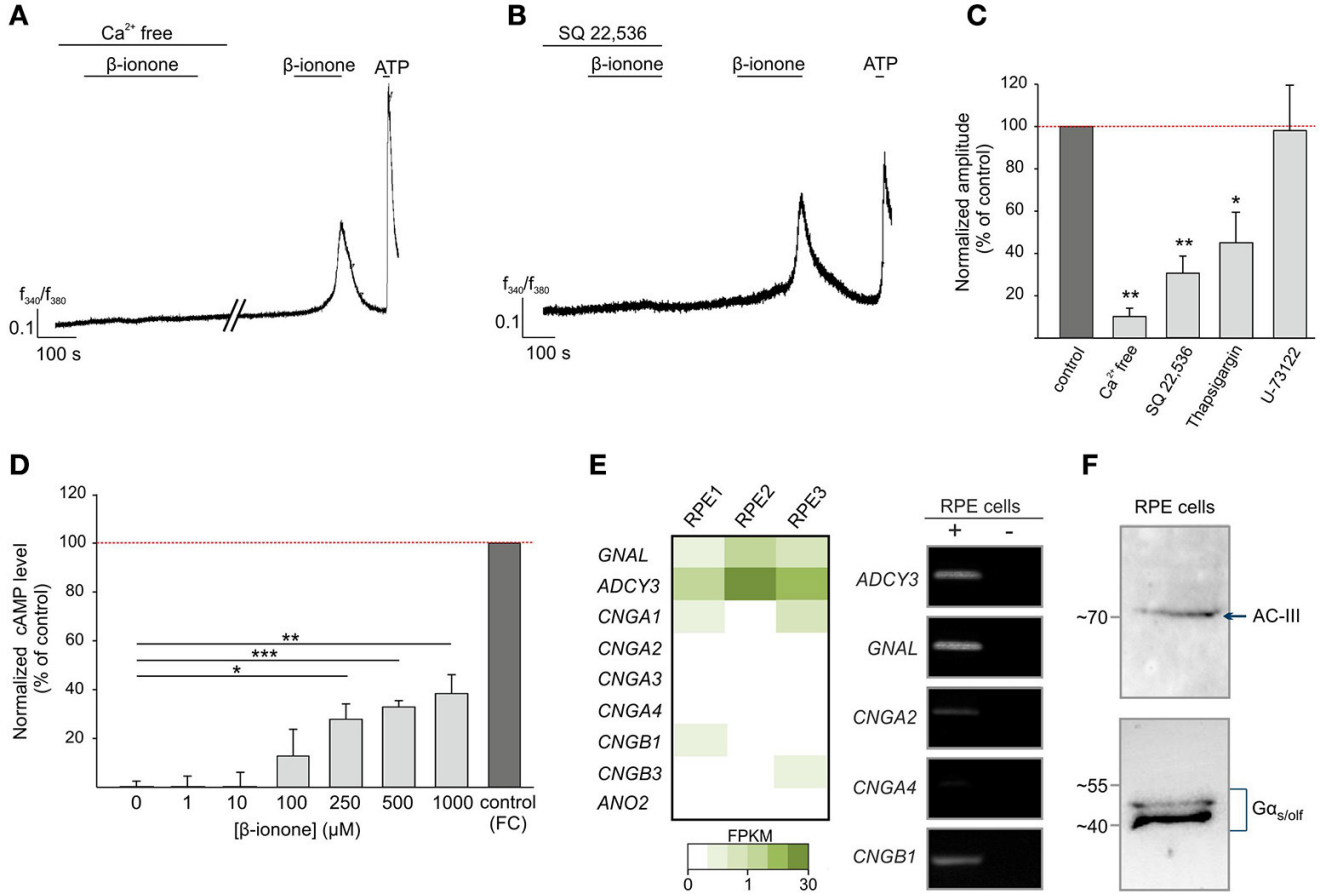
β-ionone-induced signaling in RPE cells. **(A)** Representative Ca^2+^ imaging trace of a Fura-2 loaded-RPE cell. β-ionone-induced signaling depends on extracellular Ca^2+^. β-ionone (500 μM), applied under Ca^2+^ free conditions (+1 mM EGTA), did not evoke Ca^2+^ signals. **(B)** Pre-incubation with the AC inhibitor SQ-22,536 (100 μM) abolished the β-ionone (500 μM)-induced Ca^2+^ signal in Ca^2+^ imaging experiments. **(C)** Quantification of the Ca^2+^ signal amplitudes under Ca^2+^ free conditions (*n* = 5 experiments, each with 4–9 cells), with the inhibitor SQ 22,536 (*n* = 5, each with 5–9 cells), with U-73221 (*n* = 4, each with 4–11 cells) and with thapsigargin (*n* = 4, each with 6–12 cells), which was normalized to the RPE cell responses to the control stimulations within the same experiments (β-ionone in Ca^2+^ containing buffer). **(D)** β-ionone induces an intracellular increase in the cytosolic cAMP in RPE cells after 15 min stimulation in a dose-dependent manner. The cAMP level was normalized to the AC activator forskolin (positive control) (*n* = 3). Significance was calculated by Student's *t*-test (^*^p ≤ 0.05, ^**^p ≤ 0.01, and ^***^p ≤ 0.005). **(E)** The detection of transcripts from the OR-signaling pathway components, including Gα_olf_ (*GNAL*), AC-III (*ADCY3*) and CNG channel subunits, in the RPE cells via mRNAseq and RT-PCR. **(F)** Verification of the Gα_s/olf_ and AC-III protein expression in the RPE cells by a Western blot.

We next aimed to elucidate the signaling mechanism of the β-ionone-evoked Ca^2+^ response in RPE cells. We used a Ca^2+^ free Ringer's solution to determine the origin of the agonist-evoked Ca^2+^ increase. The β-ionone-induced Ca^2+^ rise was almost completely absent after the removal of the extracellular Ca^2+^. This suggests that the odorant-evoked response of RPE cells depends on extracellular Ca^2+^ and that the Ca^2+^ released from intracellular stores does not primarily contribute to the β-ionone-induced Ca^2+^ signals (Figure 3A). Measurements with the phospholipase C inhibitor U-73122 did not reveal significant effects on the β-ionone-induced Ca^2+^ increase, whereas depletion of calcium stores by thapsigargin leads to a reduced Ca^2+^ response after β-ionone stimulation (Figure 3C, Supplementary Figure S3). The co-application of β-ionone with the adenylyl cyclase inhibitor SQ 22,536 significantly diminished β-ionone-induced Ca^2+^ responses in RPE cells (Figures 3B,C), indicating a major role of cAMP-signaling after β-ionone stimulation in this cell type. The quantification of the signal amplitudes is shown in Figure 3C. The Ca^2+^ imaging experiments suggested the involvement of cAMP. Hence, we examined whether β-ionone affects the intracellular cAMP level using a cAMP assay. We observed that the odorant application increased the cAMP level in a dose-dependent manner up to a maximal response of 40% relative to the response to the adenylyl cyclase activator forskolin, which served as a positive control (Figure 3D). In olfactory sensory neurons, ORs couple to the G_olf_ protein. This results in an activation of adenylyl cyclase III (AC-III), followed by the generation of cAMP, which, in turn, leads to the opening of the olfactory CNG channel (subunits CNGA2, CNGA4 and CNGB1) and to an influx of Ca^2+^(Mombaerts, 2004). According to our first results, we assumed that the OR51E2-initiated signal transduction mechanism in RPE cells was similar to the canonical olfactory signaling pathway. In addition, the mRNAseq analysis revealed that AC-III (*ADCY3*) and Gα_olf_ (*GNAL*) were expressed in the RPE cells (Figure 3E). The RT-PCR results verified the expression of *GNAL* and *ADCY3*, and the products Gα_olf_ and AC-III were detected at the protein level by Western blotting in RPE cells (Figures 3E,F). The mRNAseq and RT-PCR analyses showed that most of the CNG subunits were only low or not expressed in the RPE cells (Figure 3E, Supplementary Figure S4).

### Effect of the OR51E2 agonist β-ionone on proliferation and migration

**Figure 4:**
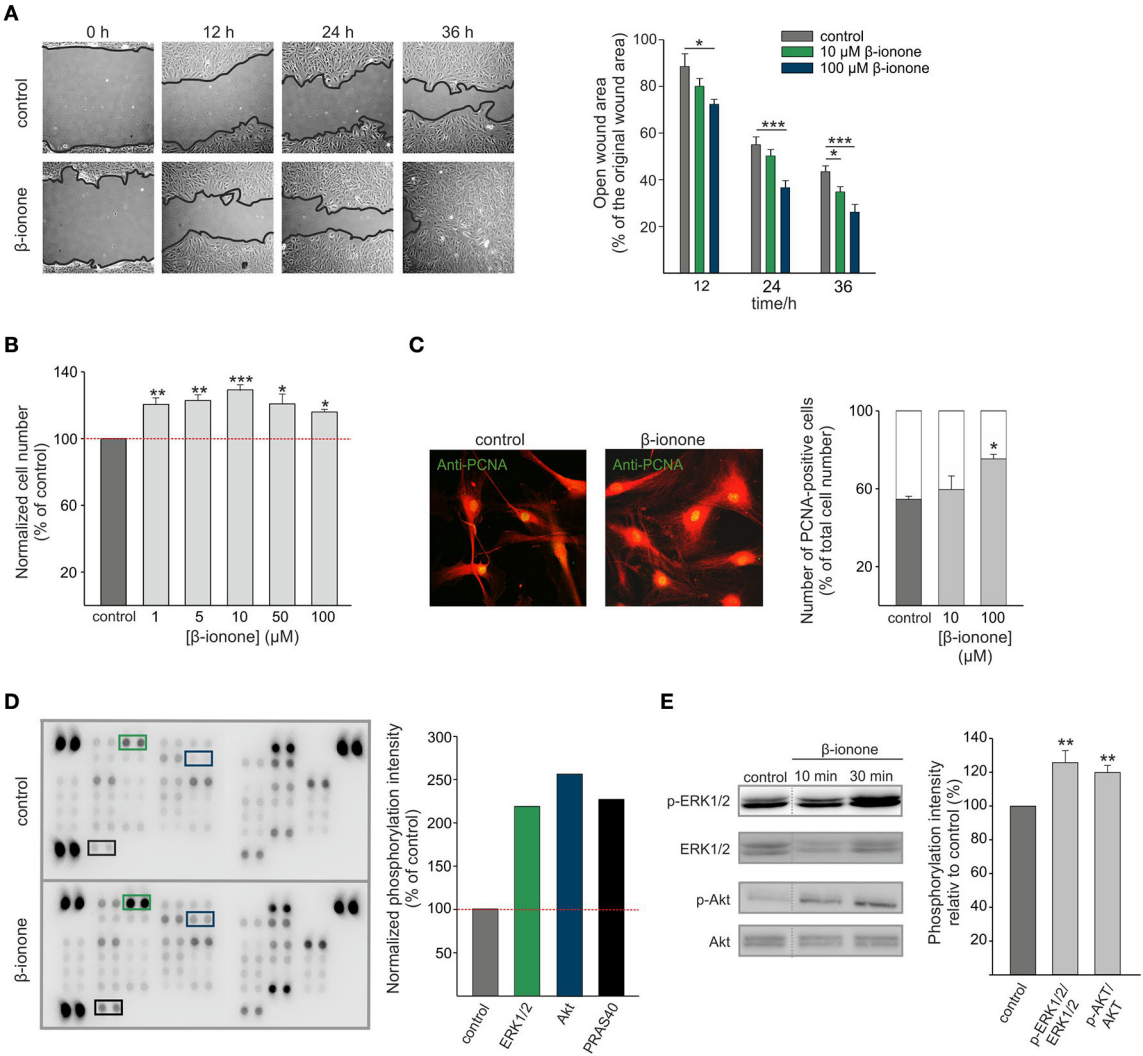
β-ionone promotes proliferation and migration of RPE cells. **(A)** A gap closure assay, using RPE cells, in the presence of 10 μM, 100 μM or 0.1% DMSO (control). The open gap area after 12, 24, and 36 h is shown relative to the original gap area at time point 0 h. The 100 μM β-ionone treatment significantly enhanced cell migration compared to the control condition (0.1% DMSO) (right and left panel). **(B)** The proliferation of the RPE cells after treatment with increasing concentrations (1 μM, 5 μM, 10 μM, 50 μM, and 100 μM) of β-ionone for 5 days compared to the control condition. The relative cell number was determined by the CyQUANT cell proliferation assay. The data are shown as the mean of four independent experiments ± the SEM with technical replicates and were normalized to the cell number in the control experiments. **(C)** Immunofluorescence confocal micrographs of the RPE cells labeled with a PCNA-specific antibody (green) and AlexaFluor 546 phalloidin (red) (left panel). Immunocytochemical staining reveals significantly enhanced numbers of proliferating cells after stimulation with β-ionone (10 μM and 100 μM) compared to the control condition (0.1% DMSO). The data are shown as the mean of three independent experiments using 100 quantified cells for each experiment, which was normalized to the cell number in the control experiments. **(D)** The OR51E2 agonist β-ionone induces the phosphorylation of protein kinases in RPE cells. The Human Phospho-Kinase Array was plotted with proteins from the RPE cells stimulated with β-ionone (500 μM; lower panel) or control (0.1% DMSO; upper panel) for 10 min. The specific antibodies against the phosphorylated protein kinases were spotted in duplicate. The colored boxes mark areas where at least a 2-fold increase in the protein signal intensities between the β-ionone-treated cells and the solvent-treated cells (control) was observed. The pixel intensities of the duplicates were averaged and β-ionone-induced phosphorylation was normalized to the control. The phosphorylation of ERK1/2, AKT and PRAS40 was enhanced relative to the control (right panel; *n* = 1). **(E)** A Western blot analysis verified the phosphorylation of ERK1/2 (T202/Y204, T185/Y187) and AKT kinases (S473) in the RPE cells after 10 and 30 min stimulations with β-ionone (500 μM) compared to stimulation with the solvent (0.1% DMSO; control) (right panel). Determination of the total amounts of the respective kinases served as controls. The mean pixel intensities of the phosphorylated proteins relative to the total protein were quantified and normalized against the control-treated cells (*n* = 4). Significance was calculated by Student's *t*-test (^*^p ≤ 0.05, ^**^p ≤ 0.01, and ^***^p ≤ 0.005).

OR51E2 is involved in the regulation of cell growth, migration and the invasiveness of skin melanocytes, melanoma cells and prostate cancer cells (Neuhaus et al., 2009; Rodriguez et al., 2014; Sanz et al., 2014, 2017; Gelis et al., 2016, 2017). Therefore, we investigated the effect of the OR51E2 ligand β-ionone on the migrative and proliferative properties of primary RPE cells. To study cell migration *in vitro*, a gap closure assay was performed. The exposure of RPE cells to β-ionone (10 μM and 100 μM) for 24 h and 36 h significantly induced the acceleration of the regeneration rate of the RPE cell monolayer compared to the control conditions (cell stimulated with solvent; 0.1% DMSO) (Figure 4A). In addition to migration, we also observed that β-ionone promoted RPE cell proliferation. RPE cells were treated for 5 days in basal medium containing different concentrations of β-ionone and the cell number was determined by measuring DNA content. Long-term β-ionone stimulation significantly increased the cell number in a dose-dependent manner, even at sub-micromolar concentrations of β-ionone. The maximal effect on proliferation (~30% increase in cell number) was noticed after a treatment with 10 μM β-ionone. This effect on the cellular proliferation rate was less pronounced at higher concentrations but was still observable compared to the control (Figure 4B). This result was confirmed via immunocytochemical staining with an antibody against the proliferating cell nuclear antigen as a marker for cell division (Figure 4C). To determine the signaling components that promotes the proliferation and migration in RPE cells, we analyzed the activation of protein kinases that mediate the regulation of various cellular processes introduced by external signals (Hecquet et al., 2002; Chan et al., 2013; Qin et al., 2013; Cheng et al., 2014; Su et al., 2014; Du et al., 2015; Wang et al., 2015). Using the Proteome Profiler Human Phospho-Kinase Array Kit we investigated the phosphorylation of 43 different protein kinases in stimulated vs. control cells. RPE cells were incubated for 30 min with β-ionone or solvent (0.1% DMSO), and the phosphorylation levels of various kinases were determined (Supplementary Figure S5). Stimulation with the odorant increased the phosphorylation of 3 kinases (ERK1/2, Extracellular-signal-regulated kinases 1/2; AKT, Protein kinase B; PRAS40, proline-rich AKT substrate 40 kDa) about the detection threshold (2-fold increase relative to control) (Figure 4D, Supplementary Figure S5). Western blot experiments were performed to confirm these results, verifying the significant enhanced phosphorylation of ERK1/2 and AKT (Figure 4E).

## Discussion

### Expression of OR51E2 in the human RPE

Comparative transcriptome analyses revealed that *OR51E2* is expressed in various human tissues, such as the prostate, colon, heart and breast (Flegel et al., 2013; Veitinger and Hatt, 2017). *OR51E2* is not only broadly expressed, but it is also one of the highest expressed ORs at the mRNA transcript level (Flegel et al., 2013). In addition, OR51E2 was also identified in epidermal melanocytes and derived melanoma cells, in which the activation of OR51E2 affects cell-type specific physiological processes, such as pigmentation and proliferation (Gelis et al., 2016, 2017). In addition to the basal layer of the epidermis, further cellular layers exist in the human body, which contain pigmented granules. These include the RPE, the iris pigment epithelium and the choroid in the human eye. In the present study, we elucidated the gene expression of *OR51E2* in pigment cells of the eye with a focus on RPE cells. The analysis of the mRNAseq data revealed the gene expression of *OR51E2* in primary RPE cells of three different donors. Interestingly, *OR51E2* showed the highest expression in adult and fetal RPE cells, whereas this receptor was not detectable in the human neural retina. The localization of OR51E2 protein was confirmed in intracellular compartments and in the plasma membrane of human RPE cells using immunocytochemical staining and Western blot analyses. These findings lead to the assumption that the receptor is present in intracellular membranes, such as endosomes, and not exclusively in the plasma membrane.

The presence of OR51E2 beyond the cytoplasmic membrane was previously demonstrated in human prostate tissue and in endosomal organelles of melanocytes (Gelis et al., 2016; Massberg et al., 2016). The intracellular localization of further ORs was also described for OR2A4, which localized to the cytokinetic structures of HeLa cells and for other ORs in sperm, ratina, heart, kidney, and brain (Zhang et al., 2012; Garcia-Esparcia et al., 2013; Kalbe et al., 2016c; Jovancevic et al., 2017a,b). Moreover, the intracellular localization and function of GPCRs in RPE cells was previously shown for the well-characterized GPCR ocular albinism type 1 (OA1), which is predominantly localized at the membranes of melanosomes, the organelles of pigment synthesis, and late endosomes/lysosomes. At the plasma membrane, only low amounts of this receptor were detected. OA1 functions in the regulation of melanosome biogenesis and the secretion of growth factors by transducing signals through the activation of heterotrimeric G proteins at the cytoplasmic side of the organelle membrane (Schiaffino et al., 1999; Schiaffino and Tacchetti, 2005; Lopez et al., 2008; Giordano et al., 2011). We assume that OR51E2 could be involved in similar processes.

Furthermore, immunohistochemical staining of retina sections, including neural retina supporting tissues (RPE/Choroid/Sclera), confirmed our OR51E2 protein expression results in the RPE and demonstrated the expression of OR51E2 in a second pigment layer, the choroid. We did not detect OR51E2 expression in the neural retina and sclera, and thus, we hypothesize that this receptor is specifically expressed in pigment cells of the human eye.

### Activation of OR51E2 in RPE cells

OR51E2 is one of the first de-orphanized human ORs. The identified carotenoid-derived volatile agonist β-ionone has a characteristic violet-like smell (Neuhaus et al., 2009). The OR51E2 agonist β-ionone is a product from the oxidative cleavage of carotenoids, such as beta-carotene and lycopene, and is catalyzed by β,β-carotene-9,10-dioxygenase 2 (BCDO2). BCDO2 belongs to the carotenoid oxygenase family β-carotene 15, 15'-monooxygenase (BCDO1), and its key function is the conversion of provitamin A carotenoids to vitamin A. Vitamin A is crucial for physiological functions, such as vision, embryonic development and cell differentiation (Amengual et al., 2013). BCDO2 expression is detected in various human tissues, such as cardiac and skeletal muscle cells, the intestine and especially in the RPE (Lindqvist et al., 2005). Thus, we assumed that β-ionone, a cleavage product of the BCDO2 enzyme, may activate intracellular localized OR51E2. The localization of OR beyond the plasma membrane was described for OR51E2 and various other ORs in diverse tissues. However, the function was only hypothesized except for OR2A4 in cells derived from cervical cancer, where participation in the cytokinesis was shown experimentally (Zhang et al., 2012). As most of the canonical downstream molecules that are involved in the GPCR signal pathway at the plasma membrane, such as the G protein, adenylyl cyclase and Ca^2+^ channels (Bootman et al., 2009; Garcia-Esparcia et al., 2013), have also been detected in intracellular compartments, the activation of a subcellular signaling cascade may be possible.

Moreover, it is conceivable that OR51E2-activating products of BCDO2 reach the RPE via the blood flow and may bind to OR51E2 at the plasma membrane. In addition, the BCDO2 enzyme is expressed in tissues that are not sensitive to vitamin A deficiency. It was therefore suggested that BCDO2 may also be involved in physiological processes other than vitamin A synthesis, pointing toward a potential biological function of the cleaved products, such as β-ionone (Lindqvist et al., 2005). Moreover, β-ionone can be found in edible and aromatic plants as well as in waters, such as lakes and rivers, due to biotransformation processes in phytoplankton (Jones, 1995; Gomes-Carneiro et al., 2006; Antonopoulou et al., 2014; Ansari and Emami, 2016). β-ionone is often used as a fragrance in cosmetics, such as perfume, soaps and shampoos (Belsito et al., 2007). Therefore, there are many opportunities for β-ionone to reach the receptor in RPE cells.

The functional role of the β-ionone-activated OR51E2 was previously described for prostate cancer cells, epidermal melanocytes and melanoma cells, whereby OR51E2 regulates the proliferation, migration, invasiveness and pigmentation (Neuhaus et al., 2009; Rodriguez et al., 2014; Sanz et al., 2014, 2017; Gelis et al., 2016, 2017). Thus, OR51E2 represents an interesting therapeutic target for the development of novel therapies to treat cancer or pigmentation disorders (Gelis et al., 2016, 2017). The activation of OR51E2 in these cells by β-ionone leads to an increase in intracellular Ca^2+^ (Neuhaus et al., 2009; Gelis et al., 2016, 2017). A β-ionone-induced Ca^2+^ response was also observed in RPE cells, but it differs in response kinetics compared to prostate cancer cells, epidermal melanocytes (time to peak: 5-10 min), as well as Hana3A cells, heterologously expressing OR51E2 (time to peak: few seconds). In the RPE cells, the maximal amplitude was reached after an average of 3 min of continuous β-ionone application, followed by a decrease in signal independent of the presence of the ligand. After reaching basal Ca^2+^ levels (after ~2 min), the cells were again responsive to odorant stimulation. The EC_50_ value of the β-ionone-induced Ca^2+^ increase in RPE cells was 91 μM. However, this is higher than in epidermal melanocytes (~60 μM), whereas the dose-response relationship in prostate cancer cells and melanoma cells was not described in the respective studies. The different response kinetics observed in Ca^2+^ imaging experiments could be a result of the different experimental conditions and initiation of different signaling pathways, which seems to depend on the cellular context in which OR51E2 is expressed. In prostate cancer cells, OR51E2 activation leads to the opening of the transient receptor potential vanilloid type 6 channel via a member of the src kinase family (Spehr et al., 2011). In melanocytes the involvement of cAMP and TRP channels as well as a partial Ca^2+^ increase from intracellular stores was suggested (Gelis et al., 2016). The β-ionone-induced Ca^2+^ rise in RPE cells is mediated primarily by cAMP as the key messenger, as shown in Ca^2+^ experiments with pharmacological inhibitors and cAMP assays and secondarily by Ca^2+^ release from intracellular stores independent of the IP_3_ pathway. In olfactory sensory neurons (OSNs), the OR-induced signal cascade also involves the activation of AC-III and the subsequent synthesis of cAMP, which in turn opens CNG channels (Nakamura and Gold, 1987; Jones and Reed, 1989; Bakalyar et al., 1990). The olfactory specific types of the G protein and AC-III were identified at the mRNA level in all analyzed samples and protein levels in RPE cells, but not all of the three CNG subtypes. The mRNAseq and PCR analysis revealed a donor dependent expression of the olfactory CNG subunits. Most of the donor samples do not express all three subunits that were necessary for the formation of a functional olfactory-type channel. Instead, we identified rod- and cone-specific CNG channel subtypes. According to our mRNAseq data, these specific subtypes are expressed only at low levels in the RPE cells (e.g., CNGA1: 0.3 mFPKM; CNGB3: 0.05 FPKM). Thus, we conclude that the β-ionone-induced pathway in RPE cells uses similar components as in OSNs with the main difference of the Ca^2+^ channel type, which mediates the observed Ca^2+^ influx. In RPE cells, we suppose that cAMP leads to the activation of protein kinase A, which in turn opens L-type Ca^2+^ (e.g., CACNA1A: 3.85 mFPKM; CACNA1A: 2.63 mFPKM) (Rosenthal and Strauss, 2002) or TRP channels (e.g., TRPM3: 1.71 mFPKM; TRPM4: 7.76 mFPKM), as shown in epidermal melanocytes and prostate cancer cells (Spehr et al., 2011; Gelis et al., 2016). Further pharmacological investigations with specific inhibitors against these components or siRNA experiments are necessary for a clear statement.

In addition to the effect on cytosolic Ca^2+^ homeostasis, β-ionone induced the activation of several downstream protein kinases. In prostate cancer cells, OR51E2 activation results in an activation of protein tyrosine kinase 2 beta (Pyk2), which in turn leads to the phosphorylation of p38 mitogen-activated protein kinases (Neuhaus et al., 2009; Wiese et al., 2015). In epidermal melanocytes, stimulation with β-ionone leads to the activation of extracellular signal-regulated kinase (ERK1/2; p42/p44 MAPK) and p38 MAPK (Gelis et al., 2016). To elucidate the effect of β-ionone on protein kinases in RPE cells, we used the Proteome Profiler Human Phospho-Kinase Array Kit to analyze, in parallel, the phosphorylation of 43 different protein kinases, and we confirmed the results via a Western blot analysis. RPE cells stimulated with β-ionone lead to the activation of ERK1/2, a finding which is consistent with the result published for skin melanocytes. Additionally, we observed an increased phosphorylation of AKT and of its substrate PRAS40 (Malla et al., 2015), which, in turn, verifies the AKT activation. An activation of protein kinases after odorant stimulation was shown in various studies (Busse et al., 2014; Maßberg et al., 2015; Kalbe et al., 2016d; Weber et al., 2017). Kim et al. were able to demonstrate that the AKT activation after stimulation with the OR10J5 agonist lyral depends on the receptor by the knockdown of OR10J5 in human umbilical vein endothelial cells (Kim et al., 2015). Key players of the OR-induced signal pathway leading to the phosphorylation of kinases were analyzed by specific inhibitors. For example, in non-small-cell lung cancer cells helional induced a Ca^2+^ signal and phosphorylation of ERK1/2 via phosphatidylinositol-4,5-bisphosphate 3-kinase, whereas in myelogenous leukemia cells the OR2AT4 ligand sandalore evoked an increase in intracellular Ca^2+^ level, which activated CaMKII and initiated ERK1/2 and AKT phosphorylation. Therefore, there are different possibilities how OR activation could lead to an enhanced protein kinase phosphorylation. We assume that, also in RPE cells, ERK1/2 and potentially AKT are activated via CaMKII or cAMP as mentioned in other studies (Bos, 2003; Dumaz and Marais, 2005; Namkoong et al., 2009). A variety of studies described the physiological effect of AKT and ERK1/2 activation in RPE cells. In both cases, the activation leads to the promotion of migration and proliferation of RPE cells (Hecquet et al., 2002; Chan et al., 2013; Qin et al., 2013; Su et al., 2014; Du et al., 2015). The effect of β-ionone on RPE cells differs from melanocytes, since AKT, but not p38 MAPK, is activated. In contrast to AKT, p38 MAPK inhibits the growth of RPE cells (Hecquet et al., 2003; Chen et al., 2016). Moreover, AKT-activation is involved in the protection of RPE cells from oxidative stress (Cheng et al., 2014; Wang et al., 2015). Furthermore, Ca^2+^ as a second messenger is involved in the regulation of RPE functions, such as proliferation, secretion of the growth factor vascular endothelial growth factor (VEGF), and pigmentation (Smith-Thomas et al., 1998; Reichhart and Strauss, 2014).

### Possible role of OR51E2 in the RPE cells

The observed effects of β-ionone on Ca^2+^ homeostasis and protein kinase activation is in accordance with the observation that a β-ionone-induced an increased proliferation and migration of primary RPE cells. The involvement of OR51E2 in the secretion process of growth factors, such as pigment epithelium-derived factor (PEDF) and VEGF, as well as in the protection from oxidative stress was investigated, but revealed no effect upon β-ionone stimulation (data not shown). A reduced proliferation rate, as a result of OR51E2 activation, was observed in melanocytes, melanoma cells and prostate cancer cells (Neuhaus et al., 2009; Gelis et al., 2016, 2017). Interestingly, stimulation of RPE cells with the OR51E2 agonist led to the opposite effect, namely, to an increased proliferation. Different physiological outcomes after activating the same OR in different cell types were previously observed for OR2AT4. The activation of OR2AT4 by its agonist sandalore results in an enhanced proliferation of keratinocytes, whereas the activation of the same receptor in chronic myelogenous leukemia cells leads to a reduced proliferation and an induction of apoptosis (Busse et al., 2014; Manteniotis et al., 2016a). An opposite physiological effect can be explained by the fact that OR activation in the respective cell type triggers different signal cascades. In RPE cells, β-ionone leads to the activation of a cAMP-mediated influx of extracellular Ca^2+^ that results in an activation of AKT-dependent signaling and finally to an increased proliferation rate. In contrast, the activation of OR51E2 in melanocytes triggers cAMP-mediated influx of extracellular Ca^2+^, as well as a release of Ca^2+^ from intracellular stores with an activation of p38 MAPK. We assume that this activation of p38 MAPK leads to the OR51E2-induced reduction of the proliferation rate in melanocytes. Therefore, the respective physiological effect depends on the cellular repertoire.

Moreover, the regulation of migration and invasiveness via OR51E2 was described in melanoma and prostate cancer cells (Sanz et al., 2014; Gelis et al., 2017). Stimulation of RPE cells with the OR51E2 agonist β-ionone led to enhanced migration, whereas the invasiveness of the RPE cells was unaffected (data not shown). The involvement of OR51E2 in the pigmentation of melanocytes was previously shown (Gelis et al., 2016). Because we observed a cell type-specific expression of OR51E2 in the investigated pigment cells of the eye, but not in neural retina and sclera, an analysis of the involvement of OR51E2 in cell pigmentation would be of interest. However, the pigmentation ability of primary RPE cells disappears during preparation and cultivation (Kernt et al., 2010). Therefore, it is not possible to analyze the role of OR51E2 in melanogenesis in primary human RPE cells. The receptor dependency of the physiological effects induced by odorants was shown in few cases by siRNA knockdown (Busse et al., 2014; Kim et al., 2015) or experiments with antagonist (Spehr et al., 2003; Neuhaus et al., 2009; Gelis et al., 2016; Kalbe et al., 2016a; Manteniotis et al., 2016a). Due to experimental difficulties, these methods could not be successfully transferred to primary RPE cells. Moreover, the analyzation of the β-ionone-induced observed pharmacological effects on human RPE cells using knockout mice was not possible. β-ionone does not activate the OR51E2 mouse ortholog Olfr78 (Pluznick et al., 2013). It is worth noting that Olfr78 knockout mice were used to demonstrate that the activation of Olfr78 with other ligands (short chain fatty acids or lactate) affects various physiological processes for example the renin secretion (Pluznick et al., 2013; Chang et al., 2015; Aisenberg et al., 2016). Aisenberg et al. could also show via CRISPR/Cas9 system that Olfr78 is involved, whereas the results with *OR51E2*-edited human airway smooth muscle cells are not clear (Aisenberg et al., 2016). In order to ensure that the observed effects of β-ionone involved the activation of OR51E2, the establishment of the CRISPR/Cas9 technology in primary human RPE cells would be necessary in future studies.

Taken together, to the best of our knowledge, we are the first that detect *OR51E2* as the most highly expressed OR in human adult and fetal RPE. Moreover, we identified OR51E2 on protein level in a further pigment cell layer of the eye, the choroid. We demonstrated that the OR51E2 agonist β-ionone induces an intracellular Ca^2+^ increase and the phosphorylation of the protein kinases AKT and ERK1/2. We also showed that adenylyl cylase and cAMP primarily mediate the Ca^2+^ increase, which indicates an involvement of an OR. Moreover, the fact that further OR51E2-activating substances evoked a Ca^2+^ response and non-activating substances had no effect on the Ca^2+^ level strengthened the assumption that OR51E2 is involved. Lastly, we provided insights into the potential role of OR51E2 in RPE cell physiology, which may be the regulation of proliferation and migration. Based on these findings, we suggest that OR51E2 acts similar to growth factor receptors in RPE cells and induces the proliferative/wound-healing responses. Therefore, we conclude that OR51E2 represents a promising therapeutic target protein for the treatment of proliferative RPE disorders, such as proliferative vitreoretinopathy (Andrews et al., 1999; Pennock et al., 2014).

## Author contributions

Wrote the paper: NJ, LG, and BK; analyzed the data: NJ, LG, HH, and GG; designed the experiments: NJ, HH, LG, MK, and AK; conducted the experiments: NJ, SK, MW, DW, AS, and GG. All authors reviewed the results and approved the final version of the manuscript.

### Conflict of interest statement

The authors declare that the research was conducted in the absence of any commercial or financial relationships that could be construed as a potential conflict of interest.
