# Supplementary material for: Odorant Receptor 51E2 Agonist β-ionone Regulates RPE Cell Migration and Proliferation
Source: Front Physiol. 2017 Nov 30;8:888. doi: 10.3389/fphys.2017.00888 (PMC5714887; doi:10.3389/fphys.2017.00888)
Supplement: Supplementary file 2 [file DataSheet1.docx]

Supplementary Material

**Odorant receptor 51E2 agonist β-ionone regulates RPE cell migration and proliferation**

**Nikolina Jovancevic^‡,^*, Soumaya Khalfaoui^‡^, Markus Weinrich^‡^, Daniel Weidinger^‡^, Annika Simon^‡^, Benjamin Kalbe^‡^, Marcus Kernt^§^, Anselm Kampik^§^, Günter Gisselmann^‡^, Lian Gelis^‡,1^, and Hanns Hatt^‡,1^**

^‡^Cell Physiology, Ruhr-University Bochum, Universitaetsstr. 150, 44801 Bochum, Germany

^§^Ophthalmology, Ludwig Maximilian University of Munich, Mathildenstr. 8, 80336 Munich, Germany.

*** Correspondence:**Nikolina Jovancevic, Department of Cell Physiology, Ruhr-University Bochum, 44801 Bochum, Germany; Nikolina.Jovancevic@rub.de

^1^equally contributing authors

# Supplementary Figures and Tables

## Supplementary Figures


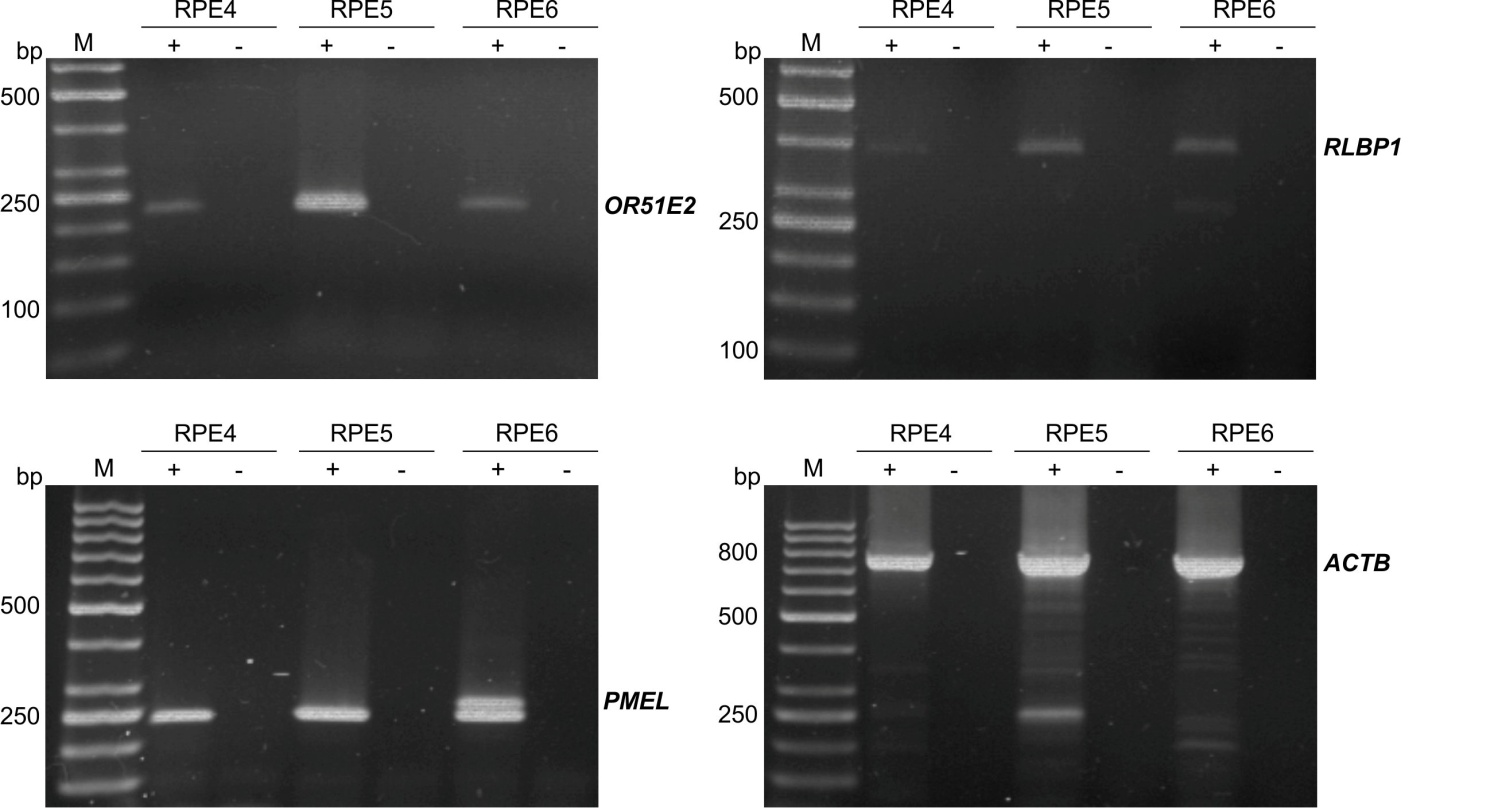


**Supplementary Figure S1.** RT-PCR validation of the RNAseq results on expression of the *OR51E2* and control genes (*PMEL*, Premelanosome protein; *RLBP1,* Retinaldehyde-binding protein 1; *ACTB*, β-actin). Gel electrophoresis of amplicons from RPE cells cDNA (+RT) and the no reverse transcriptase controls (-RT) that exclude the possibility of genomic DNA contamination of three different donors (RPE4, RPE5and RPE6). M = marker.


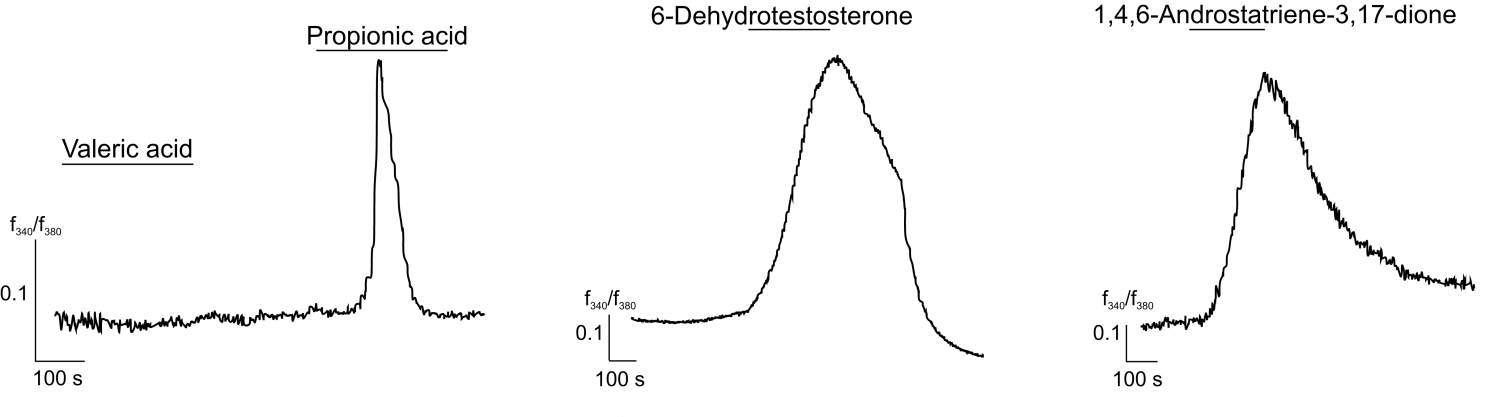


**Supplementary Figure S2.** Representative Ca^2+^ imaging traces of RPE cells stimulated with different OR51E2 agonists (1000 µM propionic acid, 100 µM 6-dehydrotestosterone and 100 µM 1,4,6-androstatriene-3,17-dione) or a OR51E2 non-activating substance (500 µM valeric acid).


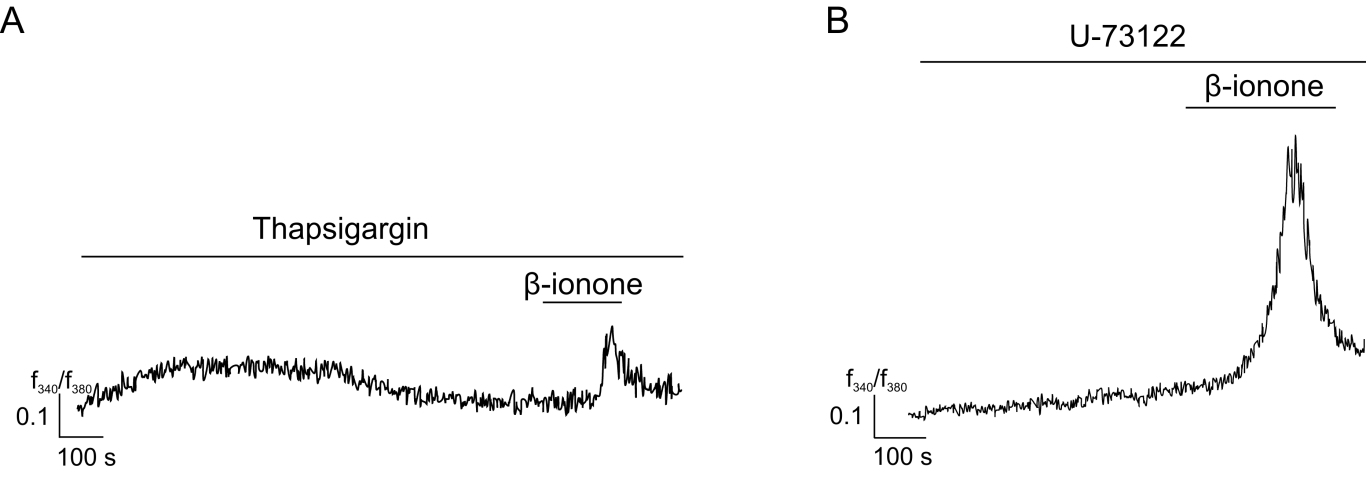


**Supplementary Figure S3.** **(A)** Representative Ca^2+^ imaging trace of a RPE cell treated with thapsigargin. After pre-incubation with thapsigargin (500 nM), the β-ionone (500 µM)-induced Ca^2+^  response was reduced **(B)** Representative Ca^2+^ imaging trace of a RPE cell treated with U-73122 (5 µM).


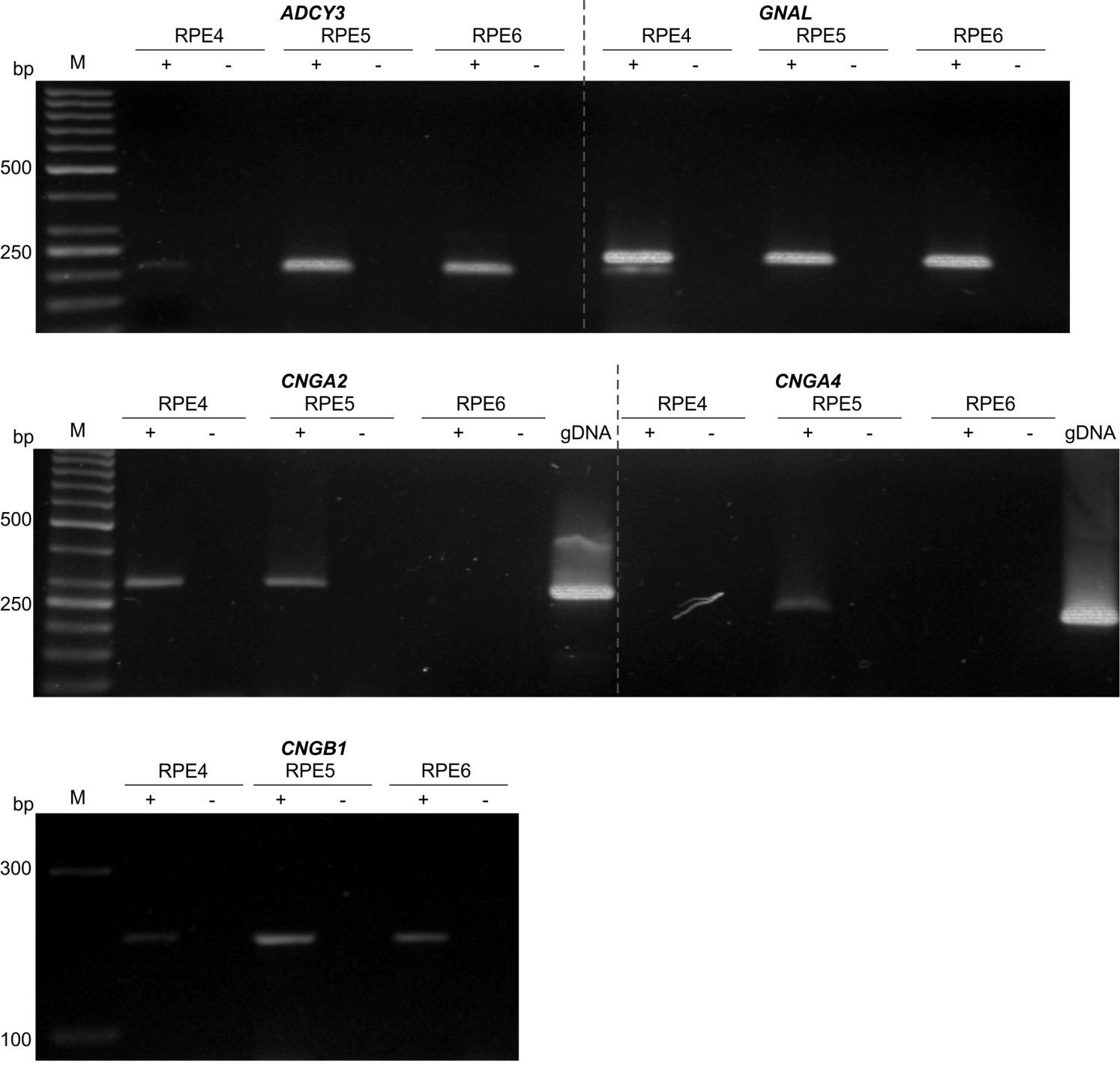


**Supplementary Figure S4.** RT-PCR validation of the RNAseq results on expression of olfactory signaling members (*GNAL*, Gα_olf_ ; *ADCY3,* AC-III and CNG channel subunits). Gel electrophoresis of amplicons from RPE cells cDNA (+RT) and the no reverse transcriptase controls (-RT) of three different donors (RPE4, RPE5 and RPE6). gDNA served as positive control. M = marker.

**
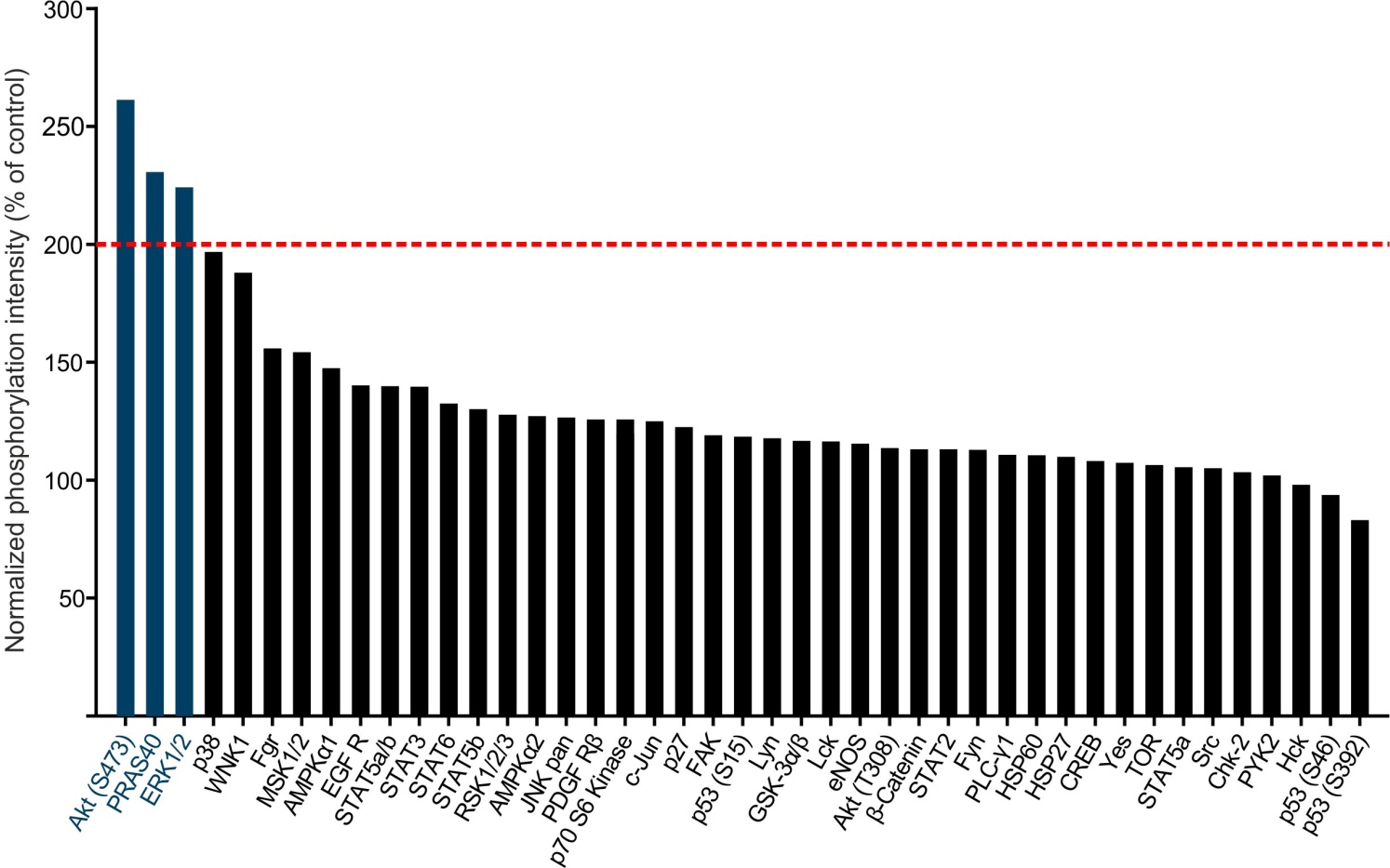
Supplementary Figure S5.** Quantification of the pixel intensities of the phosphorylated protein kinases. The pixel intensities of duplicates were averaged, and the β-ionone-stimulated RPE cells were presented relative to the control (n=1). Red dashed line represents the threshold (set at 200 % of control).

##
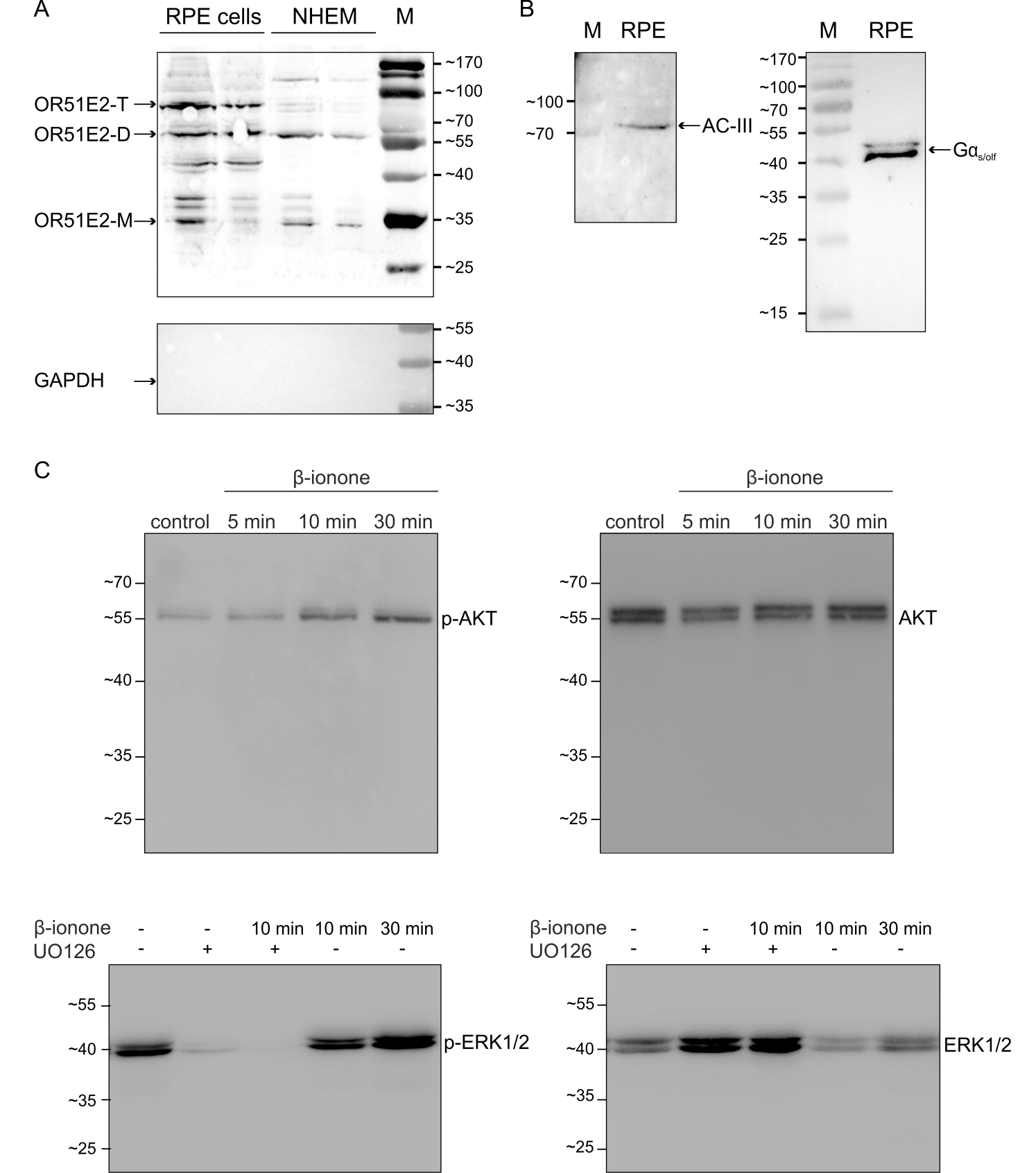


**Supplementary Figure S6.** Original entire gels. **(A)** Plasma membrane localization of OR51E2 in RPE cells and melanocytes (NHEM; control) verified by surface biotinylation and detection by Western blotting. The size of the OR51E2 monomeric (OR51E2-M) protein is 35 kDa, of the dimeric (OR51E2-D) protein is 70 kDa and trimeric (OR51E2-T) is 105 kDa. The cytosolic protein GAPDH served as a control for the enrichment of cell surface proteins and lack of cytosolic proteins. **(B)** The detection of the Gα_s/olf_ and AC-III protein expression in the RPE cells by a Western blot. **(C)** A Western blot analysis verified the phosphorylation of ERK1/2 and AKT kinases in the RPE cells stimulated with β-ionone (500 μM) compared to stimulation with the solvent (0.1 % DMSO; control). UO126, a selective inhibitor of the MAP kinase pathway, reduced the activation of ERK1/2. M = marker.

## Supplementary Table

**Supplementary Table S1.** Expression of all genes in the human RPE cells. Shown are the FPKM values for all expressed OR genes in RPE1-3. (XLSX)
